# Coxsackievirus and Adenovirus Receptor (CAR) Expression in Autopsy Tissues: Organ-Specific Patterns and Clinical Significance

**DOI:** 10.7759/cureus.37138

**Published:** 2023-04-04

**Authors:** Sudhakar Ramamoorthy, Sumit Garg, Baijayantimala Mishra, Bishan Dass Radotra, Uma Nahar Saikia

**Affiliations:** 1 Department of Pathology, NRI Medical College, Chinakakani, IND; 2 Department of Histopathology, Postgraduate Institute of Medical Education and Research, Chandigarh, IND; 3 Department of Microbiology, All India Institute of Medical Sciences, Bhubaneswar, Bhubaneswar, IND

**Keywords:** car, immunohistochemistry, rtpcr, junctional adhesion molecule, coxsackie adenovirus receptor

## Abstract

Coxsackievirus and adenovirus receptor (CAR) homologs have been identified in many species, and their proteins appeared to be highly conserved in evolution. While most of the human studies are about pathological conditions, the animal studies were more about the physiological and developmental functions of receptors. The expression of CAR is developmentally regulated, and its tissue localization is complex. Hence, we planned to study CAR expression in five different human organs at autopsy in different age groups. CAR expression was analyzed in the pituitary, heart, liver, pancreas, and kidney by immunohistochemistry, and CAR mRNA expression in the heart and pituitary by real-time PCR. In the current study, CAR expression was strong in cells of the anterior pituitary, hepatocytes, and bile ducts in the liver, acini, and pancreas and distal convoluted tubule/collecting duct in the kidney, with uniform expression in all age groups. We have noted high CAR expression in fetuses and infantile hearts, which get reduced drastically in adults due to its presumed developmental role in intrauterine life studied in animal models. In addition, the receptor was expressed in glomerular podocytes around the period of fetus viability (37 weeks) but not in early fetuses and adults. We have hypothesized that this intermittent expression could be responsible for the intercellular contact normally formed between the podocytes during the developmental phase. Pancreatic islets also showed increased expression after the emergence of the viability period but not in early fetuses and adults, which might be related to an increase in fetal insulin secretion at that particular age group.

## Introduction

Coxsackievirus and adenovirus receptor (CAR) was identified as a cell surface protein, which enables group B coxsackievirus and adenovirus to attach to the surface of cells. CAR is a type 1 transmembrane protein belonging to a subgroup of immunoglobulin superfamily [1]. CAR homologs have been identified in several species, including humans, mice, dogs, pigs, and zebrafish, and their proteins appeared to be highly conserved in evolution. While most of the human studies in CAR are about pathological conditions, the animal studies were more about the physiological and developmental functions of the receptor. The expression of CAR is developmentally regulated, and its tissue localization is complex [2]. In the adult heart, CAR is localized at cardiomyocyte sarcolemma and intercalated discs [3]. In the nervous system of vertebrates, CAR is strongly expressed during embryogenesis, followed by a drastic reduction in early postnatal stages [4]. The absence of CAR in mice results in lethality at embryonic day 11, causing heart malformations. In the adult heart, ablation of CAR results in disturbed conduction of electrical activity from the atrium to the ventricles, as indicated by prolonged PR interval in the electrocardiogram [3]. The expression of CAR is relatively reduced in all organs of adults compared to its expression in embryonic and fetal life except in polarized epithelial cells with persisting expression. However, upregulation in adulthood was noted in conditions like dilated cardiomyopathy [5] in the heart and epilepsy in the brain [6]. Though the developmental role of CAR has been studied in animals, the presence of similar functions in humans still remains unknown. In addition, only a few human studies have documented the receptor’s expression pattern in different tissues. Hence, we planned to study CAR expression in five different human organs at autopsy in different age groups, i.e., fetuses, children, and adults. The CAR expression was studied in five organs, namely the pituitary gland, heart, liver, pancreas, and kidney, to provide a compilation of different expression patterns in humans. The organs were selected based on the documented expression of CAR in animal studies.

## Materials and methods

A total of 30 consecutive autopsies of fetuses, children, and adults (10 cases from each group) were included in this study. The autopsies were performed in the Department of Histopathology at the Postgraduate Institute of Medical Education and Research (PGIMER), Chandigarh, as a routine procedure after obtaining duly informed consent from the next of kin. Institute’s Ethics Committee approved the study. All autopsy cases, including brain examinations of more than 20 weeks of gestation routinely done in the department, were included in the study. The cases with a diagnosis of dilated cardiomyopathy and coxsackievirus and adenovirus seropositivity were excluded. Fresh samples from the heart and pituitary were collected at the time of autopsy and preserved at -80℃ in RNAlater stabilization solution to analyze mRNA expression. Tissue samples from the heart, pituitary gland, kidney, liver, and pancreas were fixed in 10% buffered formalin for routine processing and H&E staining. Immunohistochemistry (IHC) was done by making a composite of these tissues. In addition, mRNA expression of CAR by both reverse transcription polymerase chain reaction (RT-PCR) and real-time quantitative polymerase chain reaction (RQ-PCR) was studied in heart and pituitary tissues collected at the time of autopsy.

Study of CAR antigen by IHC

The antibody used for IHC was CAR (H-300), a rabbit polyclonal antibody raised against amino acids 1-300 mapping at the N-terminus of CAR of human origin (Santa Cruz Biotechnology) as primary antibody and biotin-conjugated secondary antibody (Novacastra Laboratories Ltd, United Kingdom). Staining results for CAR were evaluated by estimating the percentage of cells showing specific immunoreactivity. The CAR positivity was categorized as Negative (no immunoreactivity); Weak (0-5% positive cells); Moderate (5-50% positive cells); and Strong (>50% positive cells).

The tissue samples showing moderate or strong immunoreactivity were considered positive [6]. Prostatic tissue obtained from a case of prostatic adenocarcinoma was taken as a positive control [2]. Membranous alone or membrano-cytoplasmic positivity was taken under account in pituitary (anterior pituitary) [7], liver (hepatocytes and bile ducts) [8], pancreas (pancreatic acini, intralobular and interlobular ducts, and islets) [8,9,10], and kidney (parietal epithelial cells, visceral epithelial cells, proximal convoluted tubule, and distal convoluted tubule [10]). Cardiac tissue sarcolemmal alone or sarcolemmal with sarcoplasmic CAR positivity was considered significant in cardiomyocytes [5].

Qualitative detection of CAR mRNA in heart and pituitary by RT-PCR

Heart and pituitary autopsy tissue samples were collected in RNAlater stabilization solution (Ambion, CA, USA) and stored at -80°C. RNA extraction was performed for 30 cases each (heart and pituitary), which included 10 cases from each group. RNA extraction was done from these samples by a commercially available RNA extraction kit (RNeasy Mini Kit, QIAGEN, GmbH, Hilden, Germany). After extraction, a spectrophotometer measured the RNA quantity for its purity. RNA quantity in ηg/μL and purity was measured by the ratio of absorbance at 260 nm and 280 nm. A ratio of absorbance to these two wavelengths from 1.8 to 2 indicated pure RNA. Reverse transcription (cDNA synthesis) was done using the "Revert Aid First Strand cDNA Synthesis Kit (Thermo Scientific, Lithuania)," followed by PCR. The Primers used for PCR were: CAR forward primer 5′-AGGGACCGCTGGACATCGAG-3′ and CAR reverse primer 5′-ACTCGGCCTTTCAGATCTGG-3′. In each case, positive and negative controls were run to rule out false negatives and false positives, respectively. Positive control: Hela cell line Negative control: reaction mix without template Agarose gel electrophoresis was carried out at a constant voltage of 80V. After 30 minutes of running halfway, the gel was observed under a UV trans-illuminator and documented with the Bio-Rad gel documentation system.

Semi-quantitative detection of CAR mRNA in the heart and pituitary by RT-PCR

An RQ-PCR master mix was prepared from SYBR green PCR master mix, primers, and nuclease-free water. The master mix was prepared, accounting for five additional reactions for the gene to compensate for reagents lost during pipetting. Two sets of the master mix were prepared - one for amplification of the CAR gene (test) and the other for amplification of glyceraldehyde 3-phosphate dehydrogenase (GAPDH) (internal control). The amplicon size of CAR and GAPDH were 124 bp and 100 bp, respectively. RQ-PCR was performed in a 7500 RT-PCR system as per the manufacturer's protocol (BIORAD). Amplification plots were analyzed, and CT values of CAR and GAPDH for each sample were obtained from Applied Biosystems v2.0.6 software. The RQ-PCR was performed in a 7500 RT-PCR system, amplification plots were analyzed, and CT values were obtained from Applied Biosystems v2.0.6 software. ΔCT was calculated by deducting CT of GAPDH from the CT of CAR for each sample. "Efficiency correction method" was applied, which measures the relative expression ratio from the RT-PCR efficiencies and ΔCT by using the formula: fold difference = 2-ΔCT. From the resultant values, the arithmetic mean for each group was calculated. One-way ANOVA, post hoc test, and paired t-tests were used to determine the significance between the groups.

Statistical analysis

Data were explored for any outliers, typing errors, and missing values. All quantitative variables were estimated using measures of central location (mean) and measures of dispersion (SD and standard error). All tests were two-tailed, and a p-value <0.05 was taken as significant. Statistical analyses were done using SPSS statistics v17.0 software (SPSS Inc., Chicago, Illinois, USA). The chi-square test and Fisher's exact test in 2x2 contingency tables were used to find out any significant difference in IHC CAR expression in five organs between the groups. The mean fold difference values of the heart and pituitary obtained in real-time PCR were compared between the groups using one-way ANOVA.

## Results

This was a prospective study on 30 autopsy cases divided into three groups, i.e., groups A, B, and C, with 10 cases in each group according to age: Group A: 20 weeks of gestation to 37 weeks of gestation; Group B: 37+ weeks of gestation to 12 years of age; Group C: 12 years onwards. Of these 30 cases, 20 were males and 10 were females, with a male: female ratio of 2:1. The CAR IHC positivity was assessed in five tissues, including the pituitary, heart, liver, pancreas, and kidney, on a composite formalin-fixed, paraffin-embedded (FFPE) section. Likewise, CAR mRNA expression was assessed in the heart and pituitary by both RT-PCR and RQ-PCR.

Expression of CAR in anterior pituitary

**Figure 1:**
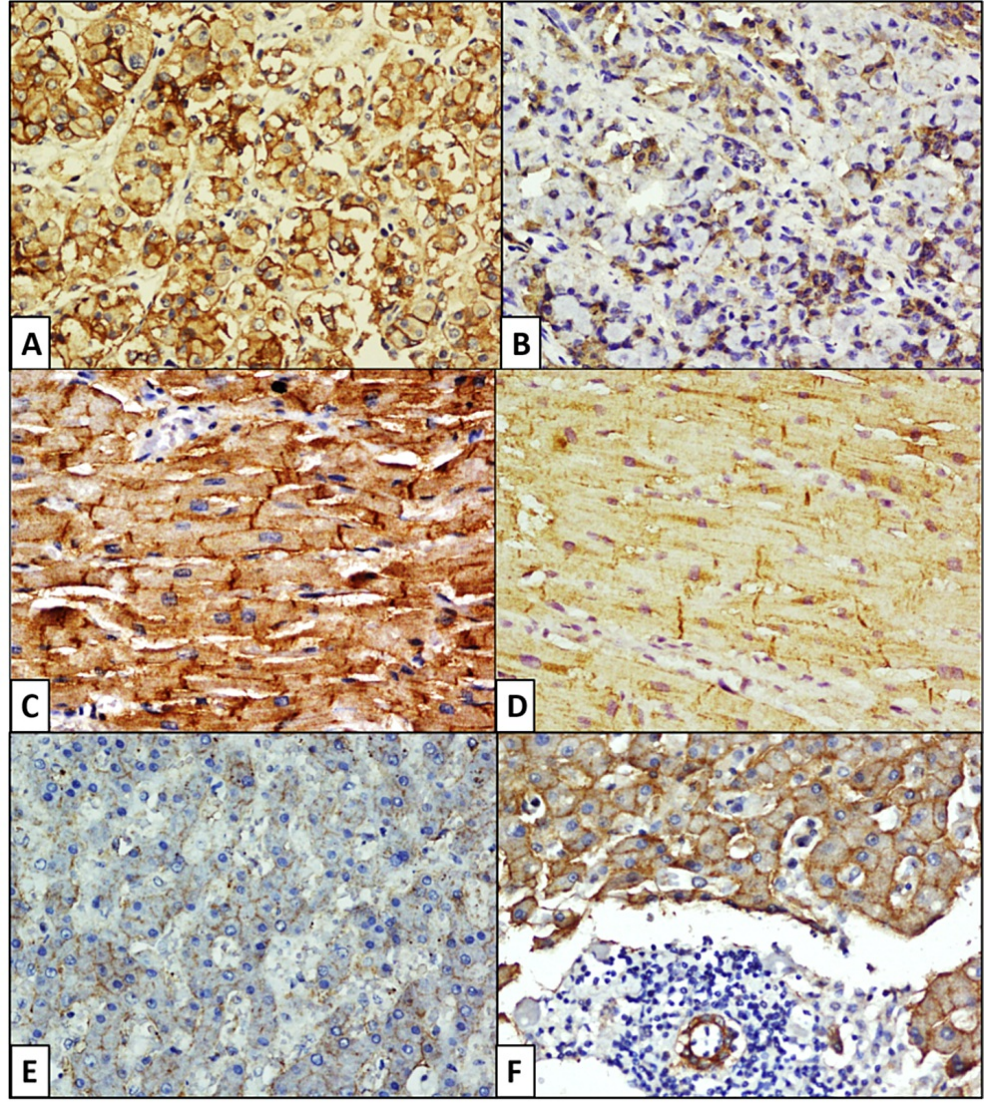
Microphotograph of anterior pituitary showing strong membranous CAR expression. A) Group C (CAR, 200X) and moderate membranous and cytoplasmic CAR expression; B) Group C case (CAR, 200X), strong CAR sarcolemmal and intercalated disc positivity in heart section, note the endothelium lining a blood vessel is negative; C) Group B case (CAR, 200X), moderate sarcolemmal CAR expression with a positive intercalated disc; D) Group B case (CAR, 200X), moderate membranous positivity of hepatocytes prominent in the canalicular surface; E) Group B case (CAR, 100X) and strong expression in hepatocytes and bile duct; F) Luminal border prominence in bile duct in a Group B case (CAR, 200X). CAR: Coxsackievirus and adenovirus receptor.

Of 30 cases, 27 (90%) showed strong CAR positivity (Figure 1A), and moderate cytoplasmic positivity was noted in three cases (10%) (two from group A and one from group C) (Figure 1B). None of the cases in any group was completely negative for the receptor. All three groups showed membranous and cytoplasmic positivity without any change in expression. Chi-square analysis did not show any significant difference (p = 0.32) among different groups.

Expression of CAR in heart

**Figure 2:**
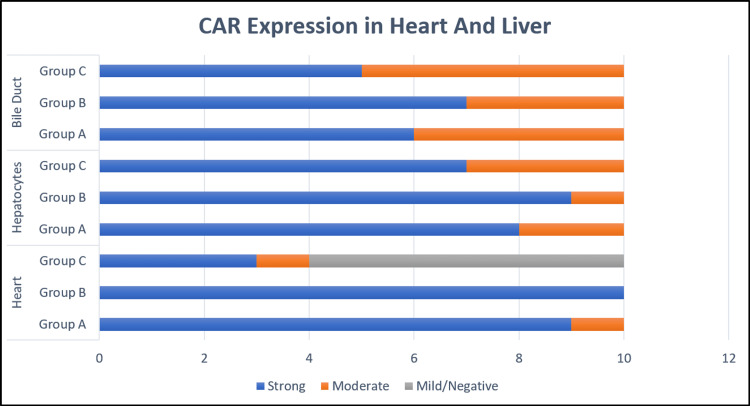
Bar diagram comparing IHC CAR expression of heart and liver tissue. IHC: Immunohistochemistry; CAR: Coxsackievirus and adenovirus receptor.

Among 30 cases, CAR was strongly expressed in nine cases (90%) of group A and 10 cases (100%) of group B (Figure 1C). Moderate expression was noted in one (10%) group A case. In group C, 60% (6/10) cases were predominantly negative, with three (30%) cases showing strong positivity and one (10%) having moderate CAR positivity (Figure 2). Of these 24 positive cases, four (16.67%) showed strong intercalated disc positivity in the cardiac tissue (Figure 1D).

Significant statistical difference was noted (p<0.001) among the groups with weak expression in group C (40%) as compared to groups A and B. The comparison plot between groups A and C, groups B and C both showed significant (p<0.001) differences; however, no significant difference was noted between groups A and B (p = 0.5). The endocardium and the blood vessels were negative for the receptor.

Expression of CAR in liver

Irrespective of age, all cases showed membranous positivity or both membranous and cytoplasmic positivity in the hepatocytes. Strong membranous positivity was noted in 80% (24/30) cases and moderate positivity in 20% (6/30) cases (Figure 2). Membranous positivity was noted more prominently on the canalicular surface than on the sinusoidal surface (Figure 1E). No difference in staining intensity was noted based on the zonal distribution of hepatocytes. Bile ducts were positive for CAR receptor in all the cases (40% moderate and 60% marked) with membranous positivity and luminal accentuation (Figure 1F). Thus, there were no significant differences in CAR expression noted either in hepatocytes (p=0.53) or bile ducts (p=0.65) among these groups. Kupffer cells were predominantly negative, with focal cytoplasmic positivity noted in only five cases (16.67%) (one case each in Group A and B and three cases in Group C). The central venules, hepatic artery branches, and portal vein radicals lacked CAR expression.

Expression of CAR in pancreas

**Figure 3:**
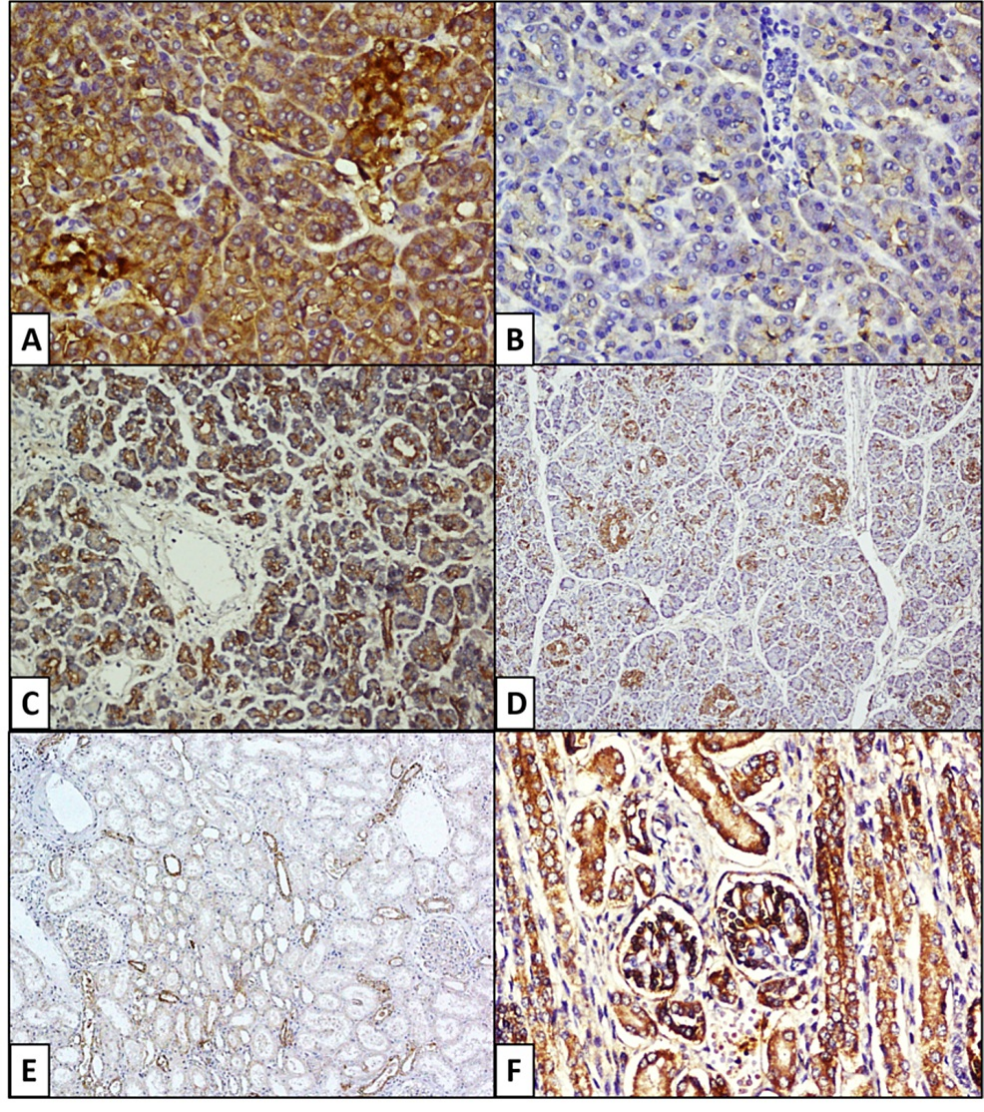
Microphotograph of pancreas showing strong membranous CAR expression in acinar cells. A) Focal strong membranous and cytoplasmic positivity in a Group C case (CAR, 200X); B) Moderate membranous expression with luminal accentuation in a group B case (CAR, 200X); C) Strong membranous expression of acinar and ductal epithelial cells along with negative stained blood vessels in a Group C case (CAR, 100X); D) Strong expression in islets and ducts and moderate expression in acinar cells (CAR, 100X); E) Kidney shows strong CAR expression in distal and in few proximal tubules whereas glomeruli are negative for the receptor expression in a group C case (CAR, 100X); F) Strong membranous CAR expression in podocytes and tubules in a group B case (CAR, 200X). CAR: Coxsackievirus and adenovirus receptor.

**Figure 4:**
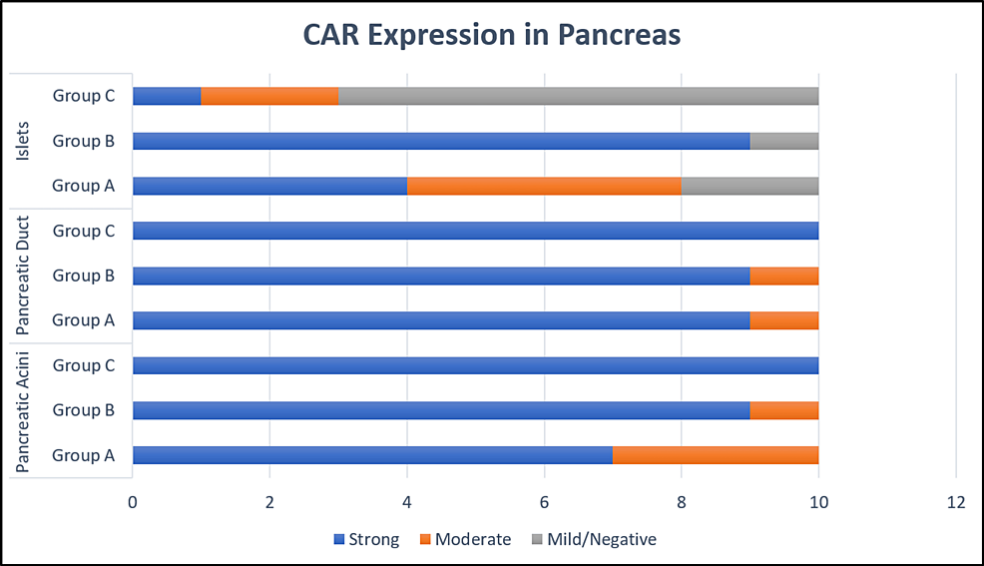
Bar diagram comparing IHC CAR expression of pancreas. IHC: Immunohistochemistry; CAR: Coxsackievirus and adenovirus receptor.

Based on MEDLINE search, CAR protein expression was evaluated in pancreatic acini, ducts (intralobular/interlobular), and islets. The acini, ducts, and islets displaying membranous staining were considered positive irrespective of the cytoplasmic pattern (Figure 3A). Among 30 cases, acini displayed moderate CAR expression in four (13.33%) cases and strong expression in the remaining 26 (86.67%) cases. Additionally, accentuation of CAR expression in the luminal border was also noted in 11 (36.67%) cases (three cases in group A and ­four cases in each groups B and C) (Figure 3B). Intralobular/Interlobular ducts showed membranous positivity in all 30 cases and strong luminal border expression in 50% of cases. However, both acini (p=0.13) and ducts (p=0.58) did not have any significant difference between the age groups. The pattern of CAR expression in acini and ducts in each group is shown in Figure 4.

**Figure 5:**
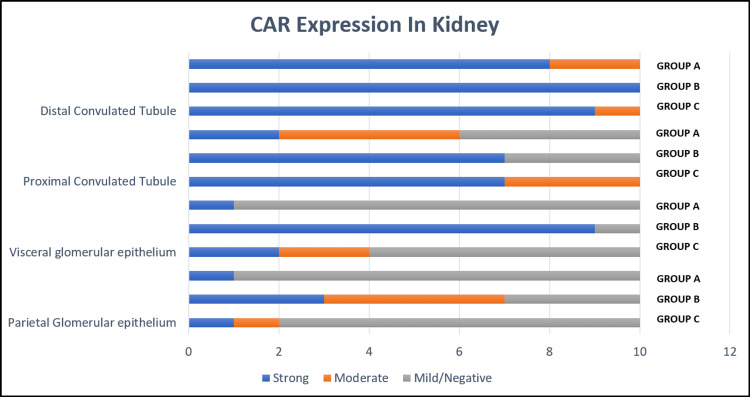
Bar diagram comparing IHC CAR expression of kidney. IHC: Immunohistochemistry; CAR: Coxsackievirus and adenovirus receptor.

The CAR expression in islet cells showed strong membranous and cytoplasmic positivity in 90% (27/30) of cases in group B (Figures 3D-5) with a significant statistical difference (p = 0.002) among the three groups. In addition, it was significant (p = 0.04) between groups A and B and highly significant (p=0.002) between groups B and C. However, the type of cell expressing CAR within the islets could not be differentiated. The endothelial cells within the lobules and interlobular septae were negative for CAR (Figure 3C).

CAR expression in kidney

In kidneys, CAR expression with cells showing membranous staining was considered positive. The parietal and visceral epithelium within the glomerulus, along with the proximal and distal convoluted tubule (PCT, DCT), were evaluated. The expression of CAR in the parietal epithelium was high in group B (80%, 8/10) as compared to group A (20%, 2/10) and group C (10%), and the difference was significant between group A and B (p = 0.02) and between group B and C (p = 0.005, Figure 5). Group B (90%, 9/10) also showed high CAR expression in the visceral epithelium as compared to group A (40%, 4/10) and group C (10%, 1/10) (Figure 3F). The Chi-square test showed a statistically significant difference between groups A and B (0.007) and groups B and C (p = 0.001). PCT and DCT/collecting ducts showed membranous CAR expression, where PCT showed 100% (10/10) positivity in group A, 70% (7/10) in group B and 60% (6/10) in group C. The difference was statistically significant between groups A and B (p = 0.032), between groups B and C (p=0.031), and between groups A and C (p=0.05). The distal tubules strongly expressed CAR (Figure 3E) in all the groups without any difference (p = 0.32, Figure 5).

CAR PCR amplification of heart and pituitary in the three different age groups

**Figure 6:**
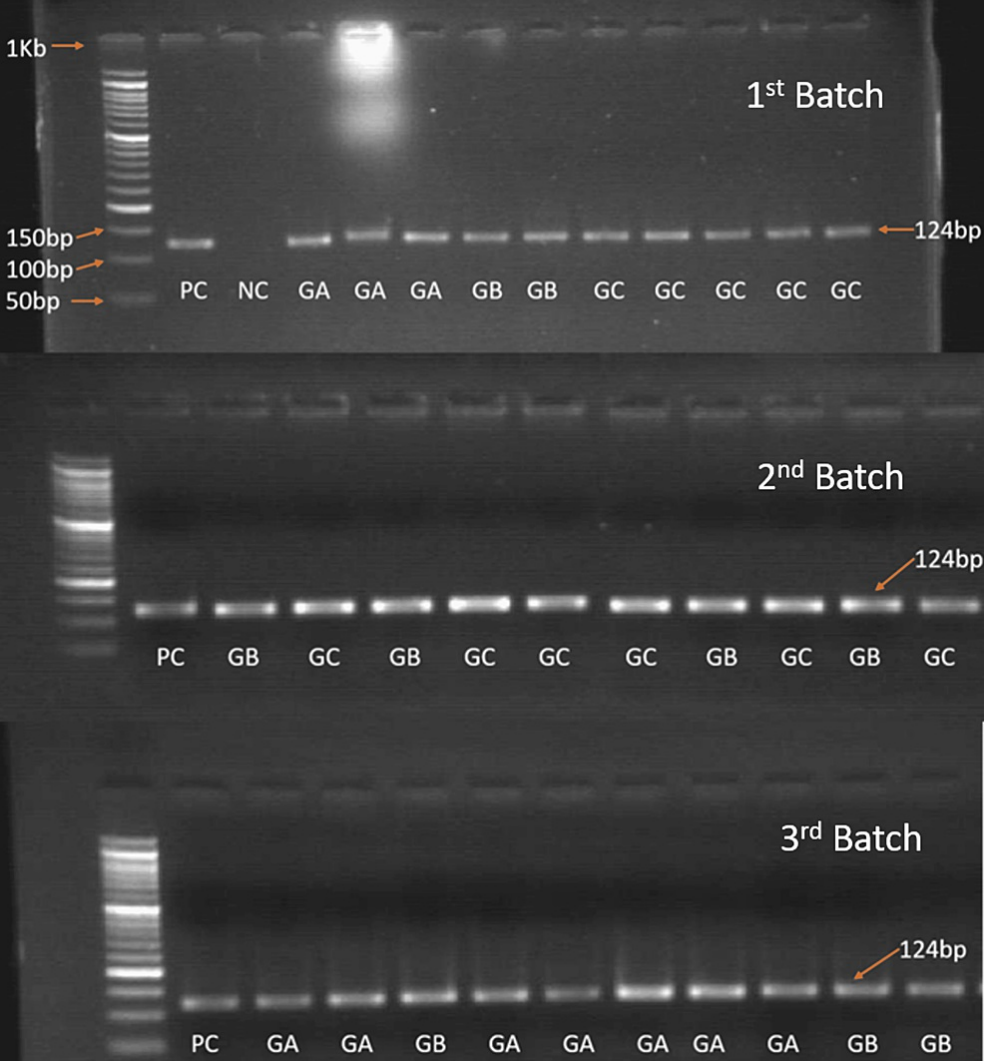
Agarose gel electrophoresis showing band positivity of CAR DNA in heart tissue in all the three groups. CAR: Coxsackievirus and adenovirus receptor.

**Figure 7:**
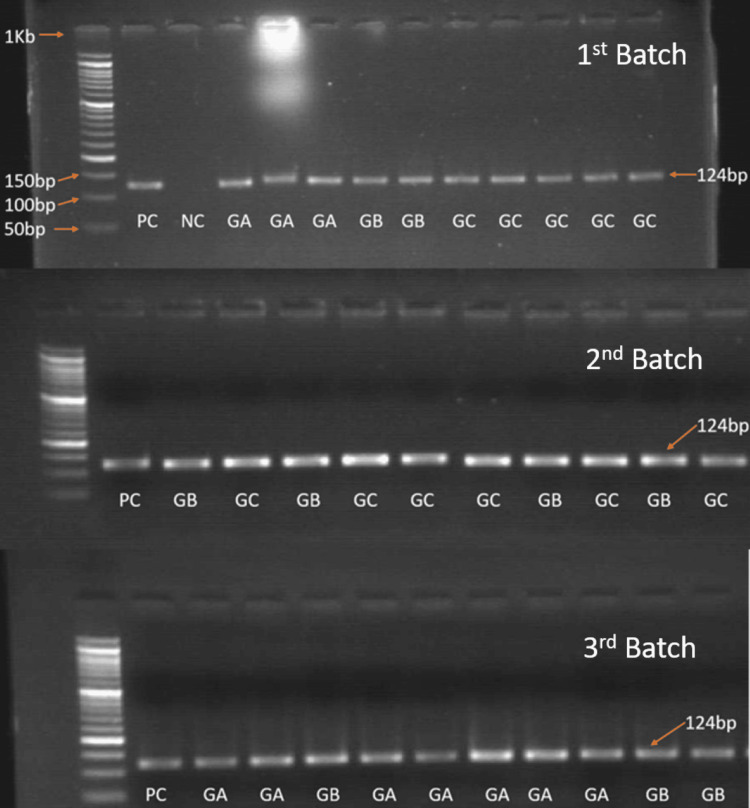
Agarose gel electrophoresis showing band positivity of CAR DNA in brain tissue in all the three groups. CAR: Coxsackievirus and adenovirus receptor.

The pituitary and myocardial tissue samples and positive control were subjected to detection of CAR by RT-PCR designed to amplify 124 bp fragment of the receptor genome. On gel electrophoresis, all test samples, including heart and pituitary, were positive for CAR genome detection (Figures 6-7). As all the samples showed positive bands, RT-PCR was done to measure the level of CAR mRNA expression relative to GAPDH.

Relative quantification of CAR gene expression in heart and pituitary by RT-PCR

We observed a positive band in all the cases (100%, 30/30) of the three groups. Thus, instead of normalizing with the housekeeping gene and measuring the difference in band intensity, a relatively more accurate RT-PCR was conducted. RQ-PCR was performed in a 7500 RT-PCR system, amplification plots were analyzed, and CT values were obtained from Applied Biosystems v2.0.6 software. From the resultant values, the arithmetic mean for each group was calculated. However, there was no significant difference obtained among the groups in heart (p=0.81) and pituitary gland (p=0.55).

## Discussion

CAR has an established role in viral attachment and internalization of group B coxsackievirus and most of its subgroups (except B) of adenoviruses. The CAR expression profile in different tissues was also reported to have a critical role in tissue susceptibilities to adenovirus-based gene therapy [11]. Its physiological function of being a component of the tight junction complex and involvement in cell-to-cell interaction of all epithelial cells was later identified. While the developmental function of the receptor was studied in murine models, rats, and zebrafish, information about the receptor's biological function is still sparse and sometimes contradictory. There are studies in the heart and brain of murine models where CAR was found to be highly expressed in embryonic and fetal life and reduced drastically after birth. However, the expression of CAR and its developmental role in human tissues is still lacking. On an extensive MEDLINE search, we could not find any human studies about an age-specific expression of CAR receptors in tissues other than the heart and brain. The CAR expression in pathological conditions has been studied in humans. This was a pilot study to see an age-specific expression of CAR receptor in human tissues for which five organs (pituitary gland, heart, liver, pancreas, and kidney) were selected, which also have been studied for CAR expression earlier in animal models [5,8,12,13]. Our study showed strong CAR expression in the anterior pituitary in 100% of cases and complete loss of its expression in the posterior pituitary with no significant difference (p = 0.32). A handful of studies have reported CAR expression in the brain regarding its developmental role [14] and expression in pathological conditions [7,14,15]. These findings are similar to a study by Persson A et al., where marked staining positivity was noted in the choroid plexus and anterior pituitary [7]. However, we observed (n=30) that CAR expression was relatively more at both proteins as well as in mRNA level compared to the previous study (n=4). There was no significant difference between the groups (p = 0.55) with relative quantification of CAR mRNA expression in the pituitary. This could be explained as the anterior pituitary being composed of adenotype cells, which have intercellular contacts, and no difference in expression was noted in the age groups. The receptor could possibly have only cell adhesion function in the anterior pituitary rather. However, its developmental role in the pituitary needs to be explored in embryonic life. In addition, CAR expression in the anterior pituitary is important in gene therapies for brain tumors such as neuroblastoma and medulloblastoma [14]. The adenovectors used in cancer gene therapy that acts through CAR may bind to normal tissues of high CAR expression, like the anterior pituitary causing hormonal imbalance.

The IHC of the heart showed strong expression in fetuses and infants, while the expression was significantly reduced in adults. Only 40% (4/10) of cases in group C showed receptor expression with a highly significant difference (P<0.001) from groups A and B. The finding was supported by a similar study conducted in murine models [16] and rats [17], where high CAR expression was observed in embryos and fetuses, with a reducing trend in postnatal life and very low expression in adults [16]. Dorner AA et al. showed that cardiac deletion of CAR before the embryonic day 11.5 led to mortality of mice which histologically showed disorganization of myofibrils. In addition to myofibril disorganization, loss of CAR in the embryonic heart also causes hyperplastic left ventricles and sinuatrial valve abnormalities [16-18]. Lim BK et al. proposed that CAR was responsible for the localization of connexin 45 at the AV node and of β-catenin and ZO-1 at the intercalated discs in mice [19] which is necessary for AV node conduction. However, we did not include cases of myofibrillar degeneration, including all types of cardiomyopathy (CMP), as previous studies have shown the re-expression of CAR in human adult hearts [5]. This re-expression has also been described in adult rats in cases of myocardial infarction [19].

The re-expression of CAR helps in tissue remodeling by regulating the hyperproliferation of cardiomyocytes secondary to myocardial injury. However, there is a lack of enough evidence of CAR expression in myocardial infarction (MI) in humans. The CAR expression in DCM cases is more diffuse compared to MI, where the expression was confined to the infarcted zone [20]. Additionally, both these conditions express CAR in areas of angiogenesis with capillary proliferation suggesting tissue repair. On the contrary, we did not observe endothelial positivity for CAR in normal other than DCM and MI, nor any positivity in the subendothelial layer of the vessel wall was noted, which has been reported in 20 adult hearts by Noutsias M et al. [5]. In addition, 4/24 (16.67%) cases also showed intercalated disc positivity, similar to previous studies [21]. The intercalated discs contain gap junction, adherens junction, and desmosomes which have been linked to cardiac arrhythmia in animals and human models. Proteins of adherens junction and desmosomes affect conduction indirectly through gap junctions [21]. Along with protein expression, we confirmed CAR mRNA expression by RT-PCR, which showed 124 bp band positivity in all the cases irrespective of groups. However, semi-quantitative RT-PCR did not show any significant difference among the groups (heart p = 0.81). This could be due to a low sample size. Immunohistochemical analysis of CAR in the liver showed 80% (24/30) strong and 20% (6/30) moderate positivity within hepatocytes in all the groups. The non-parenchymal cells, including endothelial cells and kupffer cells, did not show any significant CAR expression. In addition, kupffer cells expressed cytoplasmic positivity in five (16.66%) cases which included 30% of group C, 10% of group A, and 10% of group B. This was in contrast to the study by Yu Q et al., who reported that adenoviral transduction was more efficient in non-parenchymal cells with increased expression of CAR, responsible for viral entry compared to parenchymal cells [22]. In addition, CAR expression in hepatocytes was more accentuated on the canalicular surface than the sinusoidal surface [23]. This finding was confirmed in our study as well, where canalicular surface accentuation of CAR was present in all age groups, while the bile ducts showed 60% (18/30) strong and 40% (12/30) moderate expression of CAR. However, this expression in hepatocytes (p=0.53) and bile ducts (p=0.65) was not statistically significant among various groups. This uniformity in expression assures that CAR functions only as an adhesion molecule rather than having any developmental role in the liver. However, the expression of CAR in kupffer cells in present and previous studies remains unexplainable. Being a component of the reticuloendothelial system, CAR positivity within the cytoplasm of kupffer cells could be due to the phagocytosis of soluble CAR isoforms. Further studies are necessary to quantify the receptor's expression in the liver as it may hinder the transduction efficiency of adenovector to target organs in gene therapy.

The pancreas showed strong CAR expression both in acini and ducts in all groups, similar to Mena I et al. This could possibly be the reason why coxsackie B virus infection frequently causes pancreatitis, and CVB has been implicated in 20-34% of pancreatitis cases [9]. Additionally, Mena I et al. also showed a loss of CAR receptors and reduced CAR mRNA levels in islets. However, we found strong CAR expression in islets, which was significantly higher in group B compared to groups A and C (A and B: p=0.04; B and C: p=0.002). Studies have shown that intercellular contacts are necessary for proper secretory response by the endocrine pancreas [24], and tight junctions may start developing as early as after 30 weeks of gestation. This could be one of the possible reasons for initiating insulin secretion at this age, explaining our finding of high CAR expression in islets in group B compared to group A. However, the low CAR expression in group C is unexplainable. Hodik M et al. showed that CAR protein is expressed in islets of Langerhans in a higher percentage of individuals with type I diabetes (T1D) or at risk of T1D than non-diabetic individuals [25]. Thus, there could be an association of CBV infection with an increased risk of developing T1D later in life. In the kidneys, we found a significant difference in CAR expression among the groups in both parietal epithelium and visceral epithelium and within the glomerulus. The conversion process from vesicle to maturation stage happens during 20-35 weeks of fetal life, and intercellular contact is present around 35-40 weeks of gestation [26]. This could be the reason for significant (p=0.001) CAR expression in group B (>37 weeks of gestation) as compared to groups A and C in our study. The parietal epithelium also showed significantly higher CAR expression in group B (p=0.01) than in other groups. Akin to bile ducts in liver and pancreatic ducts, the CAR receptor was found to be strongly expressed in convoluted tubules, especially DCT and collecting tubules. However, there were seven cases of tubular necrosis with CAR negativity which explains the negative expression of CAR in these cases. Thus, the statistically significant expression of CAR in PCT has to be considered with caution requiring more studies to confirm or exclude this finding. This suggests that intermittent CAR expression could be responsible for intercellular contact generally formed between the podocytes during the developmental phase. In addition, we observed persistent CAR expression at all ages in the anterior pituitary, hepatocytes, bile ducts, pancreatic acini, and pancreatic ducts. An increased expression in pancreatic islets might be related to an increase in fetal insulin secretion at that particular age group. In addition, adenoviral vectors and CAR have been widely utilized in cancer gene therapy, rapidly developing areas in pre-clinical and clinical cancer research. In addition, adenovirus of type 5 (Ad5) is most frequently used in several types of cancer gene therapy, such as gallbladder, brain, colon, rectum, lung, prostate, liver, stomach, ovary, esophagus, and peritoneum. Cancer gene therapy redirects cytotoxic T cells to recognize specific antigens on cancer cells, which can be achieved by a chimeric antigen receptor-like CAR or a modified T-cell receptor [27]. Several authors have suggested that engineered T cells can also be used in heart diseases. Aghajanian H et al. demonstrated the efficacy of redirected T-cell immunotherapy to specifically target pathological cardiac fibrosis in mice models. They concluded that the adoptive transfer of T cells that express a chimeric antigen receptor against fibroblast activation protein results in a significant reduction in cardiac fibrosis and restoration of function after injury [28].

Limitations of the study

The study may have potential limitations. The sample size of the study was small; hence generalizability and external validity of the findings is to be confirmed with future studies of a larger sample size.

## Conclusions

This was a pilot study, the first of its kind, where age-specific CAR expression in the heart, pancreas, and kidneys was studied. CAR is a homotypic cell adhesion receptor involved in cell-to-cell interaction and cancer gene therapy. We suggest that intermittent CAR expression could be responsible for intercellular contact during the developmental phase. An increased expression in pancreatic islets might be related to increased fetal insulin secretion in that particular age group. A high CAR expression in cardiac tissue of fetuses and infants, which is reduced drastically in adults, may be useful in analyzing its therapeutic or prognostic role in various cardiac diseases, including DCM. Besides, the role of CAR in cancer gene therapy can be exploited for various cancers.

## References

[1] Philipson L, Pettersson RF (2004). The coxsackie-adenovirus receptor--a new receptor in the immunoglobulin family involved in cell adhesion. Curr Top Microbiol Immunol.

[2] Forrest JC, Campbell JA, Schelling P, Stehle T, Dermody TS (2003). Structure-function analysis of reovirus binding to junctional adhesion molecule 1. Implications for the mechanism of reovirus attachment. J Biol Chem.

[3] García-Becerril GE, Cruz-Montalvo AE, De La Cruz MA (2019). Differential expression of coxsackievirus and adenovirus receptor in endomyocardial tissue of patients with myocarditis. Mol Med Rep.

[4] Honda T, Saitoh H, Masuko M (2000). The coxsackievirus-adenovirus receptor protein as a cell adhesion molecule in the developing mouse brain. Brain Res Mol Brain Res.

[5] Noutsias M, Fechner H, de Jonge H (2001). Human coxsackie-adenovirus receptor is colocalized with integrins alpha(v)beta(3) and alpha(v)beta(5) on the cardiomyocyte sarcolemma and upregulated in dilated cardiomyopathy: implications for cardiotropic viral infections. Circulation.

[6] Anders M, Vieth M, Röcken C (2009). Loss of the coxsackie and adenovirus receptor contributes to gastric cancer progression. Br J Cancer.

[7] Persson A, Fan X, Widegren B, Englund E (2006). Cell type- and region-dependent coxsackie adenovirus receptor expression in the central nervous system. J Neurooncol.

[8] Korn WM, Macal M, Christian C (2006). Expression of the coxsackievirus- and adenovirus receptor in gastrointestinal cancer correlates with tumor differentiation. Cancer Gene Ther.

[9] Mena I, Fischer C, Gebhard JR, Perry CM, Harkins S, Whitton JL (2000). Coxsackievirus infection of the pancreas: evaluation of receptor expression, pathogenesis, and immunopathology. Virology.

[10] Chehadeh W, Kerr-Conte J, Pattou F, Alm G, Lefebvre J, Wattré P, Hober D (2000). Persistent infection of human pancreatic islets by coxsackievirus B is associated with alpha interferon synthesis in beta cells. J Virol.

[11] Horwood NJ, Smith C, Andreakos E, Quattrocchi E, Brennan FM, Feldmann M, Foxwell BM (2002). High-efficiency gene transfer into nontransformed cells: utility for studying gene regulation and analysis of potential therapeutic targets. Arthritis Res.

[12] Zanone MM, Favaro E, Ferioli E (2007). Human pancreatic islet endothelial cells express coxsackievirus and adenovirus receptor and are activated by coxsackie B virus infection. FASEB J.

[13] Raschperger E, Neve EP, Wernerson A, Hultenby K, Pettersson RF, Majumdar A (2008). The coxsackie and adenovirus receptor (CAR) is required for renal epithelial differentiation within the zebrafish pronephros. Dev Biol.

[14] Fuxe J, Liu L, Malin S, Philipson L, Collins VP, Pettersson RF (2003). Expression of the coxsackie and adenovirus receptor in human astrocytic tumors and xenografts. Int J Cancer.

[15] Bissel SJ, Winkler CC, DelTondo J, Wang G, Williams K, Wiley CA (2014). Coxsackievirus B4 myocarditis and meningoencephalitis in newborn twins. Neuropathology.

[16] Dorner AA, Wegmann F, Butz S (2005). Coxsackievirus-adenovirus receptor (CAR) is essential for early embryonic cardiac development. J Cell Sci.

[17] Kashimura T, Kodama M, Hotta Y (2004). Spatiotemporal changes of coxsackievirus and adenovirus receptor in rat hearts during postnatal development and in cultured cardiomyocytes of neonatal rat. Virchows Arch.

[18] Chen JW, Zhou B, Yu QC (2006). Cardiomyocyte-specific deletion of the coxsackievirus and adenovirus receptor results in hyperplasia of the embryonic left ventricle and abnormalities of sinuatrial valves. Circ Res.

[19] Lim BK, Xiong D, Dorner A (2008). Coxsackievirus and adenovirus receptor (CAR) mediates atrioventricular-node function and connexin 45 localization in the murine heart. J Clin Invest.

[20] Tatrai E, Bedi K, Kovalszky I (2011). No mutation but high mRNA expression of Coxsackie-Adenovirus Receptor was observed in both dilated and ischemic cardiomyopathy. Forensic Sci Int.

[21] Lisewski U, Shi Y, Wrackmeyer U (2008). The tight junction protein CAR regulates cardiac conduction and cell-cell communication. J Exp Med.

[22] Yu Q, Que LG, Rockey DC (2002). Adenovirus-mediated gene transfer to nonparenchymal cells in normal and injured liver. Am J Physiol Gastrointest Liver Physiol.

[23] Ashworth MA, Leach FN, Milner RD (1973). Development of insulin secretion in the human fetus. Arch Dis Child.

[24] Collares-Buzato CB, Carvalho CP, Furtado AG, Boschero AC (2004). Upregulation of the expression of tight and adherens junction-associated proteins during maturation of neonatal pancreatic islets in vitro. J Mol Histol.

[25] Hodik M, Anagandula M, Fuxe J (2016). Coxsackie-adenovirus receptor expression is enhanced in pancreas from patients with type 1 diabetes. BMJ Open Diabetes Res Care.

[26] Quaggin SE, Kreidberg JA (2008). Development of the renal glomerulus: good neighbors and good fences. Development.

[27] June CH, O'Connor RS, Kawalekar OU, Ghassemi S, Milone MC (2018). CAR T cell immunotherapy for human cancer. Science.

[28] Aghajanian H, Kimura T, Rurik JG (2019). Targeting cardiac fibrosis with engineered T cells. Nature.

